# The Comparison of Intraocular Pressure Measured by Transpalpebral Method Using Diaton and Rebound Tonometry by iCare in the Eyes Before and After Transepithelial Photorefractive Keratectomy (TPRK) in Saudi Arabia

**DOI:** 10.7759/cureus.33031

**Published:** 2022-12-28

**Authors:** Sultan Alzuhairy

**Affiliations:** 1 Department of Ophthalmology, College of Medicine, Qassim University, Buraydah, SAU

**Keywords:** eye care, tprk, diaton, tpiop, icare

## Abstract

Introduction: Intraocular pressure (IOP) measurement is vital to select and monitor a patient undergoing ocular surgery. The validity of tonometry by independent researchers is useful. In this paper, we compare intraocular pressure (IOP) by rebound tonometry using iCare (Tiolat Oy, Helsinki, Finland) with transpalpebral IOP (tpIOP) method by using Diaton (Bicom Inc., NY, USA) before and after transepithelial photorefractive keratectomy (TPRK) in Saudi Arabia.

Methods: This cross-sectional validity study was held at a private ophthalmology hospital in central Saudi Arabia from January 2021 to February 2022. The tonometry was performed before, at week 1 (W1), and month 1 (M1) after TPRK. The tpIOP and IOP by iCare were compared using matched-pair analysis. The agreement in IOP by two methods was reviewed using the Bland-Altman plot. Central corneal thickness (CCT), spherical equivalent (SE) before surgery, and gender were correlated to the difference in IOP by two tonometers. The main outcome was the difference in IOP measured by Diaton and iCare.

Results: We studied 202 eyes of 101 patients. The median difference in IOP by Diaton and iCare was -1.0 mmHg before, at W1, and M1 follow-ups. Before surgery, tpIOP by Diaton was 15.0±2.8 mmHg and by iCare was 16.0±3.7 mmHg (P<0.001). At W1, tpIOP was 15.9±2.5 mmHg and 16.9±3.4 mmHg by iCare (P<0.001). At M1, tpIOP was 15.7±4.1 mmHg and 16.5±5.4 mmHg by iCare (P<0.001). IOP by two methods was within ±2 mmHg in 73.3%, 69.8%, and 75.2% of the eyes before, at W1, and M1 of TPRK. Pre-CCT (P<0.001) was the significant predictor of the difference in IOP by two methods at W1 and M1 (P=0.001). iCare gave the overestimation of IOP compared to Diaton in 18.3%, 22.8%, and 17.8% of the eyes before, W1, and M1 follow-ups.

Conclusions: IOP by iCare and Diaton was similar. Central corneal thickness was the predictor of IOP differences by tonometers.

## Introduction

Intraocular pressure (IOP) measurements using some newer tools are based on the principles of rebound tonometry, transpalpebral tonometry, handheld applanation tonometry, pneumotonometry, noncontact ocular response analyzer, and resonant applanation tonometry. Old methods such as Schiotz indentation tonometry are rarely used anymore. However, applanation tonometry using a Goldmann tonometer (GAT) that is mounted on a slit lamp biomicroscope is used with the patient in a sitting position and remains the gold standard for IOP measurement [1,2]. Self-measurement of IOP by glaucoma patients, task shifting of IOP measurement to mid-level eye-care professionals, and self-assessment by glaucoma patients require a tonometer that is reliable, fast, and easy to use; does not require an anesthetic agent; and can be used with the patient in different positions (sitting and supine) [3-5]. Each new tool has its benefits and disadvantages. Therefore, the selection of a tonometer to screen and monitor IOP in their patients is a dilemma for eye-care providers.

Diaton (Bicom Inc., NY, USA), a transpalpebral, transscleral IOP measuring tool, is noted as being on par with the GAT [6,7]. Other researchers have noted that transpalpebral IOP (tpIOP) measured by Diaton is good for screening the IOP of young healthy persons but is not reliable for monitoring IOP in the eyes with glaucoma or thin corneas [8,9]. iCare (Tiolat Oy, Helsinki, Finland) is a rebound tonometer that provided repeatable IOP measurements that matched IOP measured by the Perkins tonometer [10]. It is easy to use and preferred by eye patients for its comfort compared with GAT [11].

Because myopia in the younger population is at epidemic proportions and many choose not to wear contact lenses and spectacles, the demand for refractive surgeries is increasing [12]. Glaucoma in an otherwise healthy myopic eye can negatively influence the outcome of refractive surgery. Therefore, IOP measurement before refractive surgery is a part of patient selection. Even after surgery, IOP needs to be monitored, and if high, prompt intervention is needed [13]. The findings on the reliability of a tonometer to measure IOP in myopic patients undergoing refractive surgeries are inconclusive, as are recommendations for its use over GAT [14,15].

Single-step transepithelial photorefractive keratectomy (TPRK) is an accepted surgical procedure to address myopia in young adults. In this procedure, both epithelium and subepithelial stroma are ablated by laser. In other corneal surgeries, the flap of epithelium was lifted, and laser surgery was done on stroma to correct myopia. Although surgical outcomes at three months after surgery are similar, TPRK had epithelial thinning and reduced central corneal thickness (CCT) if measured soon after surgery [16].

The literature demonstrates the effectiveness of both the Diaton and iCare tonometers compared with GAT [4,10,17]. To the best of our knowledge, the effectiveness and reliability of Diaton compared with iCare, especially in the eyes subjected to transepithelial photorefractive keratectomy (TPRK), have not been examined. We compare IOP measured by Diaton and iCare 200 in myopic eyes before and after TPRK and the determinants of differences in IOP measured by the two tonometers.

## Materials and methods

The regional research and ethics committee of Qassim University, Saudi Arabia, approved this study (607/43/6830). The tenets of the Helsinki Declaration were strictly followed. We evaluated the eyes undergoing TPRK to treat myopia among patients at a private clinic in central Saudi Arabia from January 2021 to February 2022. Those consenting to participate were included in the study. We excluded patients undergoing refractive surgery by methods other than TPRK. This was a cross-sectional comparison study.

To calculate the sample for this study, we assumed that in a population of 1000 eyes treated with TPRK for myopia in our institute, 80% would have a 2 mmHg difference in IOP measured by Diaton and iCare as documented by Sánchez Pavón et al. [18]. To achieve a 95% confidence interval (CT) with a 5% acceptable error margin, we needed to review IOP using both methods in 198/200 eyes. We used OpenEpi software to calculate the sample size [19].

One ophthalmologist measured IOP. Mid-level eye-care professionals assisted in the ocular assessment of these patients. The demographic information included age in years, gender, and eye operated on. If both eyes were to be operated on, the IOP of one randomly selected eye was first measured with the iCare (Tiolat Oy, Helsinki, Finland) tonometer and then with the Diaton (Bicom Inc., NY, USA). In the fellow eye, the Diaton was used first, followed by the iCare. If only one eye of a patient was to be operated on by TPRK, we used the tonometers in random order.

iCare 100 is a reliable IOP measurement device that allows taking single or rapid six-series measurements [20]. Its advanced positioning assistant enables correct alignment. The red or green light of a probe base indicator indicates the correct or incorrect positioning of the device. Diaton tonometer measurements were performed with the patient in a sitting position and gazing down at a 45° angle; the tonometer was placed in contact with the eyelid at the superior limbus. The device was activated when the signaling mechanism indicated the correct vertical position. There was a five-minute interval between the iCare and Diaton measurements [21].

To determine myopia status, spherical and cylindrical refraction in diopters was noted. The spherical equivalent (SE) of myopia was defined as spherical+(cylinder/2) refraction in diopters. Myopia was further graded as “mild” if the spherical equivalent (SE) was ≤3.00 Ds, “moderate” if SE was between -3.00 and -6.00 Ds, and “severe” if SE was >6.00 Ds [22].

Central corneal thickness (CCT) and epithelial corneal thickness (epiCT) were measured at baseline with the help of anterior optical coherent tomography (Pentacam AXL, Oculus, Germany) [23]. The participants were further grouped based on CCT. Grade I comprised CCTs of <530 µm, grade II had CCTs between 530 and 560 µm, and those with CCTs of >560 µm were in grade III [24].

We used SCHWIND AMARIS 1050RS (SCHWIND eye-tech-solutions GmbH, Kleinostheim, Germany) for transepithelial laser ablation (1.3 seconds/diopter). To create aspheric ablation, we applied SmartPulse allocation software. After applying topical anesthetic, the standard procedures were used for laser application. The corneal epithelium was not removed with a blade. The surgery steps adopted were like those described in the literature [25,26].

Postoperative care included topical steroid application. We routinely followed up on patients at week 1 (W1) and month 1 (M1). CCT and IOP were measured using both tonometers at follow-up visits once the absence of epithelial defect was confirmed. An IOP of less than 22 mmHg was labeled normal. If the IOP was 22 mmHg or more, we considered it a high pressure and treated the patient with glaucoma medications.

The data were collected from hospital records using Microsoft Excel® (Microsoft® Corp., Redmond, WA). The data were cleaned, and the information on the eye as a unit was compiled. Then, the data were transferred into the Statistical Package for Social Sciences (SPSS) (IBM SPSS Statistics, Armonk, NY) version 25 spreadsheet. Both univariate and multivariate analyses were conducted. The qualitative data were presented as numbers and percentage proportions. The quantitative data, if distributed normally, were presented as mean and standard deviations. The difference in IOP using Diaton and iCare was our main outcome at baseline and at W1 and M1 follow-ups. To study the efficiency of iCare compared to Diaton, we used the Bland-Altman plot graph of SPSS. We considered a difference in IOP using two methods of less than 2 mmHg to be an acceptable difference. If the IOP differed by more than 2 mmHg, we considered it an overestimation or an underestimation. We considered the measurement by Diaton as reference since this was a transpalpebral method and is less likely to be affected by TPRK. We correlated the qualitative variables of IOP measured by Diaton and iCare by matched-pair analysis to estimate the two-sided P value of correlation. A P value of <0.05 was considered statistically significant.

## Results

**Figure 1:**
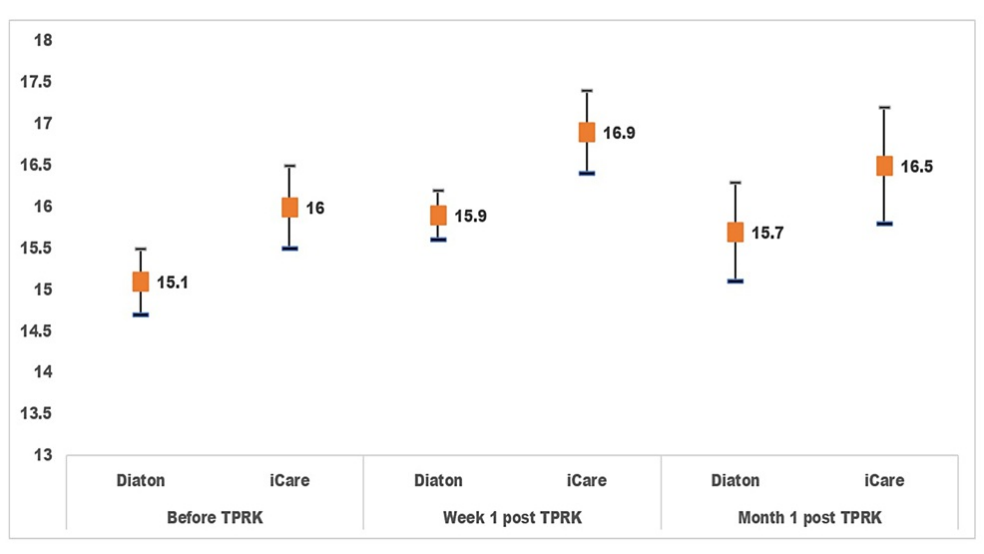
Intraocular pressure measured before, at week 1, and month 1 after transepithelial photorefractive keratectomy (TPRK) by Diaton and iCare The x-axis shows intraocular pressure (IOP) measured at a different time in relation to TPRK. The y-axis shows intraocular pressure (mmHg). The red squares indicate the mean value of IOP in 202 eyes. The upper and lower ends of the vertical line indicate the 95% confidence interval of the mean IOP

We examined 202 eyes of 101 patients. Of the participants, 47 were male, and 54 were female. The mean age of participants was 25.7±5.7 years (range: 18 to 42). Based on SE, 93 eyes (43%) had mild myopia, 79 (39.1%) had moderate myopia, 21 (10.4%) had severe myopia, and nine (4.5%) had astigmatism (>1 D). The CCT was grade 1 (<530 µ) in 68 (33.7%) eyes, grade II (530-560 µ) in 71 (35.1%) eyes, and grade III (>560 µ) in 63 (31.2%) eyes. The mean SE of 202 eyes was -3.2±1.9 D (range: -8.0 to 0.5). The mean CCT was 545.2±38.2 µ (range: 404 to 609). The mean epithelial corneal thickness was 56.5±7.6 µ (range: 15 to 79). The profile of intraocular pressure measured by Diaton and iCare for all eyes and those in subgroups before, at W1, and at M1 is presented in Table 1 and Figure 1.

**Table 1:** Intraocular pressure by Diaton tonometer and iCare tonometer before and after transepithelial photorefractive keratectomy (TPRK) SD, standard deviation; IOP, intraocular pressure; CCT, central corneal thickness

Before		IOP by Diaton		IOP by iCare		Validation
		Mean	SD	Mean	SD	
Gender	Male	15.0	2.8	15.4	3.4	P=0.06
	Female	15.1	2.8	16.6	3.8	
CCT grade	Less than 530	14.5	2.8	14.8	3.2	P<0.001
	530-560	14.9	2.6	15.3	2.9	
	More than 560	15.9	2.9	18.2	3.5	
Myopia grade	Mild	15.1	2.8	16.0	3.5	P=0.834
	Moderate	15.0	2.8	16.1	4.2	
	Severe	15.2	2.7	16.1	2.9	
	Astigmatism	14.8	3.8	15.4	3.2	
Week 1 after TPRK						
Gender	Male	15.6	2.8	16.2	3.2	P=0.074
	Female	16.2	2.7	17.4	3.6	
CCT grade	Less than 530	15.4	1.9	15.1	2.8	P<0.001
	530-560	15.5	3.4	16.4	5.4	
	More than 560	16.4	2.5	18.2	3.5	
Myopia grade	Mild	16.1	2.2	17.1	2.9	P=0.506
	Moderate	16.0	2.6	17.0	4.0	
	Severe	14.7	3.0	15.1	3.5	
	Astigmatism	16.1	2.1	16.9	2.6	
IOP before	<15 mmHg	15.2	2.2	16.0	3.2	P=0.485
	≥15 mmHg	16.4	2.4	17.5	3.5	
IOP week 1 after surgery	<15 mmHg	13.1	1.0	14.8	3.1	P=0.02
	≥15 mmHg	17.1	1.9	17.7	3.2	
Month 1 after TPRK						
Gender	Male	15.0	3.2	15.6	4.9	P=0.296
	Female	16.3	4.7	17.3	5.7	
CCT grade	Less than 530	14.9	5.0	14.9	5.8	P=0.004
	530-560	15.5	2.6	16.4	5.4	
	More than 560	16.8	3.7	18.2	3.5	
Myopia grade	Mild	16.3	5.1	17.5	6.5	P=0.509
	Moderate	15.1	3.1	15.2	3.9	
	Severe	14.7	1.9	16.7	4.3	
	Astigmatism	17.3	2.8	17.3	4.8	
IOP before	<15 mmHg	15.1	4.7	16.2	5.5	P=0.182
	≥15 mmHg	16.2	3.6	16.8	5.3	
IOP week 1 after surgery	<15 mmHg	14.6	4.0	15.6	5.3	P=0.534
	≥15 mmHg	16.2	4.1	16.9	5.4	

The tpIOP after TPRK increased at W1 and then declined at M1. IOP measured by iCare was significantly higher than that noted by tpIOP before surgery. However, the changes in IOP measured by iCare did not vary significantly by time. The mean pair tpIOP and iCare IOP difference was -0.955 (95% CI: -1.38 to -0.54) before TPRK, -0.95 (95% CI: -1.34 to -0.56) at W1 post TPRK, and -0.812 (95% CI: -1.23 to -0.4) at M1 post TPRK.

The tpIOP before surgery was median 1.0 mmHg (interquartile range {IQR}: -2.0 to 0.0) (minimum-maximum: 12.0; +8.0) more compared with iCare IOP. One week after TPRK, the median difference was -1.0 (IQR: -2.0 to +1.0) (minimum-maximum: -9.0; +8.0), and one month after TPRK, it was -1.0 (IQR: -2.0 to +1.0) (minimum-maximum: -14.0; +10). At W1 post TPRK, the difference in IOP measured by the two methods was not significantly higher (P=0.983), but at M1 compared with before TPRK, it was 1.77 mmHg higher using Diaton than iCare compared with the IOP difference noted in same eye before surgery (P<0.001). The difference between tpIOP and IOP using iCare and determined by qualitative and quantitative variables is presented in Table 2.

**Table 2:** Difference in intraocular pressure measured by Diaton tonometer and iCare tonometer before and after transepithelial photorefractive keratectomy (TPRK) 95% CI, 95% confidence interval; tpIOP, transpalpebral intraocular pressure

Qualitative variables	Before TPRK			Post TPRK week 1			Post TPRK month 1		
	Paired difference of mean	95% CI	P value	Paired difference of mean	95% CI	P value	Paired difference of mean	95% CI	P value
Gender	-2.49	-2.93 to -2.05	<0.001	-2.48	-2.89 to -2.08	<0.001	-2.35	-2.77 to -1.92	<0.001
Pre-central corneal thickness (CCT) grades	5.46	541 to 551	<0.001	5.28	523 to 533	<0.001	-5.46	540 to 551	<0.001
Type of refractive error	-2.68	-3.12 to -2.26	<0.001	-2.68	-3.1 to -2.28	<0.001	-2.4	-2.9 to -1.9	<0.001
Quantitative variables	Before TPRK			Post TPRK week 1			Post TPRK month 1		
	Pearson coefficient (r)		P value	Pearson coefficient (r)		P value	Pearson coefficient (r)		P value
Age	0.097		0.17	-0.013		0.86	0.193		0.006
Spherical equivalent	0.019		0.788	0.011		0.88	0.019		0.785
Central corneal thickness	-0.258		<0.001	-0.328		<0.001	-0.229		<0.001
tpIOP before	0.2		0.004	-0.075		0.29	0.013		0.85
tpIOP 1 week post-surgery				0.152		0.031	-0.004		0.96
tpIOP 1 month post-surgery							-0.117		0.096

Gender and the type of refractive error were negatively correlated with the difference in tpIOP and iCare IOP before and after TPRK. CCT had a significant and positive correlation with the difference between tpIOP and iCare IOP. Age and the difference of IOP at M1 were significantly and positively correlated. tpIOP measured at W1 post-TPRK was positively correlated with the difference in IOP using the two methods.

**Figure 2:**
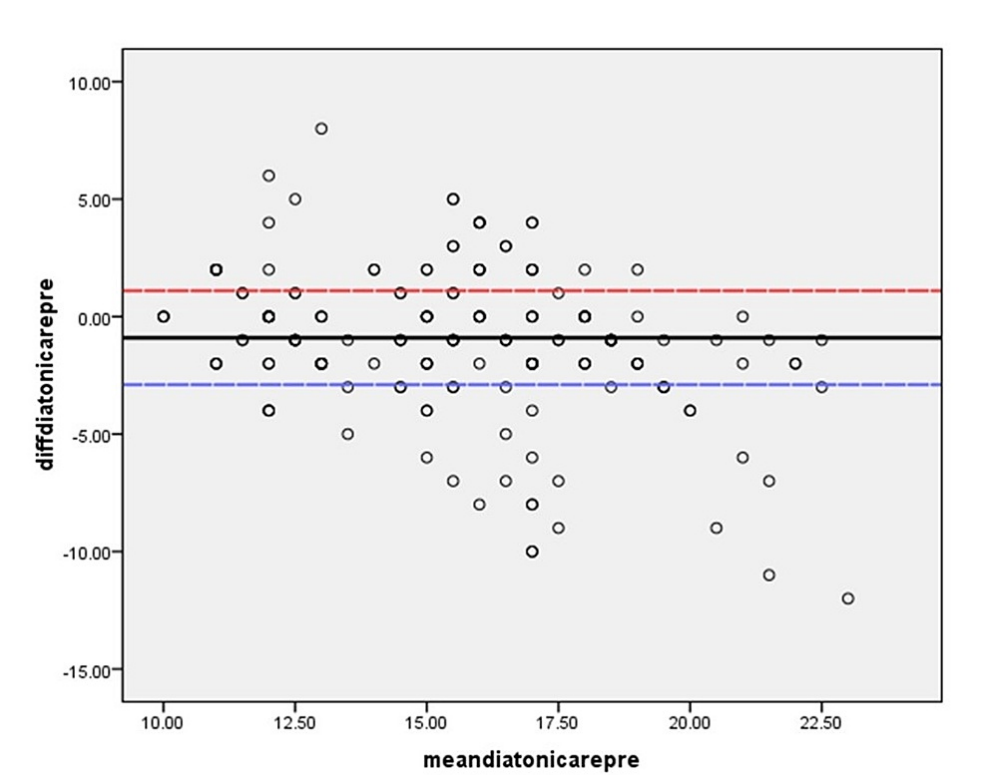
Bland-Altman plot of IOP measured by Dalton and iCare before transepithelial photorefractive keratectomy (TPRK) The black line is the mean difference in IOP by two methods. The blue line depicts the lower end of two standard deviations. The red line depicts the upper end of two standard deviations IOP: intraocular pressure

**Figure 3:**
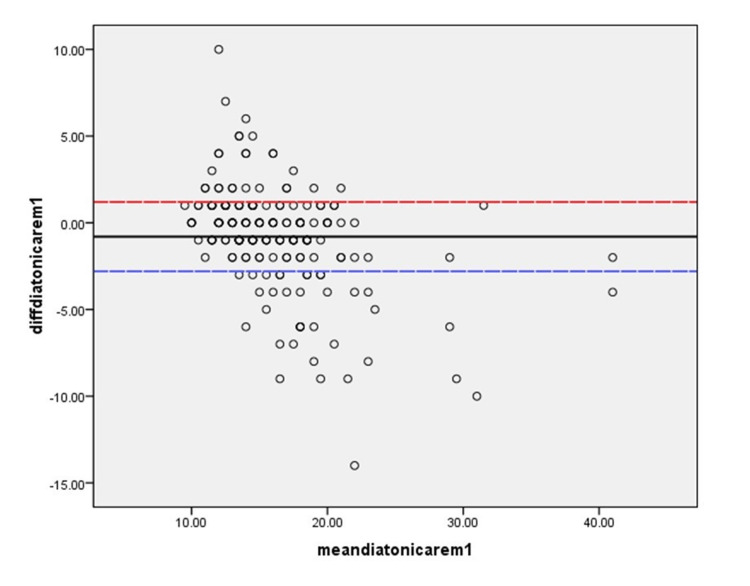
Bland-Altman plot of IOP measured by Dalton and iCare one week after transepithelial photorefractive keratectomy (TPRK) The black line is the mean difference in IOP by two methods. The blue line depicts the lower end of two standard deviations. The red line depicts the upper end of two standard deviations IOP: intraocular pressure

**Figure 4:**
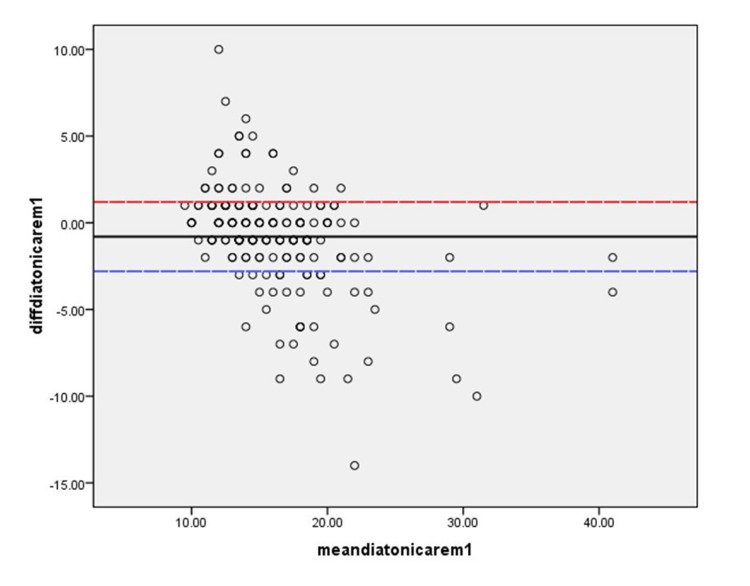
Bland-Altman plot of IOP measured by Dalton and iCare one month after transepithelial photorefractive keratectomy (TPRK) The black line is the mean difference in IOP by two methods. The blue line depicts the lower end of two standard deviations. The red line depicts the upper end of two standard deviations IOP: intraocular pressure

Linear regression analysis revealed that the difference in tpIOP and iCare IOP at one month after TPRK can be predicted (F=10.0, CCT before TPRK {standardized beta=-0.235, t=-3.47, and P=0.01}, and age {standardized beta=0.103, t=2.95, and P=0.04}). The consistency of IOP using the two methods is shown in a Bland-Altman plot before TPRK, at W1, and at M1 after TPRK (see Figures 2-4).

Measuring IOP using two methods was within ±2 mmHg in 73.3%, 69.8%, and 75.2% of the eyes before, at W1, and at M1 of TPRK. Pre-CCT (P<0.001) was a significant predictor of differences in IOP using the two methods both at W1 and M1 (P=0.001). iCare gave an overestimation of IOP compared with Diaton in 18.3%, 22.8%, and 17.8% of the eyes before, at W1, and at M1 follow-ups.

## Discussion

Among newer tools to measure IOP in the young myopic population, we found that IOP measured by iCare was higher than that measured by Diaton before and after TPRK. However, the difference in IOP measured using the two methods was not statistically significant. The CCT significantly influenced the difference in IOP using Diaton and iCare both before and after TPRK. A thinner cornea after TPRK at week 1 and month 1 had a greater difference in IOP measured by Diaton compared with iCare and compared to that before surgery. One month after TPRK, the difference in IOP using the two tonometers was significantly more in older patients than in younger patients.

Although IOP measured by these two tonometers was similar in our study, one should be careful in selecting a tonometer to measure IOP in the eyes with thin corneas. With an adequate sample size, the randomization of the eyes to be tested first by one tonometer and then an adequate interval using the other tonometer, and standard analysis methods, the present study shows true outcomes with less influence of bias. Both Diaton and iCare have been found to be user- and patient-friendly [9,27]. Their efficiency compared to GAT in previous studies suggested that they are a good tool for screening IOP in healthy people, but Diaton fell short in monitoring IOP following refractive surgeries for myopia, especially in the eyes with thin corneas [14]. iCare was shown to be more reliable than GAT post-LASIK surgeries [20].

We noted that at one week following TPRK, the IOP measurements by Diaton and iCare increased and then declined one month after TPRK. Thus, either tonometer was good to monitor IOP one week after TPRK. However, after one month, the IOP difference measured by the two tonometers was significant; hence, the monitoring of IOP should be done by the same type of instrument, and we recommend that clinicians not switch from Diaton to iCare or vice versa.

CCT was a predictor of the difference in IOP measured by Diaton and iCare before and after TPRK in our study. Diaton provided a lower IOP than iCare in the eyes with thinner corneas. This was also noted by Rozhdestvenskaya et al. [14], when they compared tpIOP with IOP measured by GAT. Toker et al. also noted the influence of CCT on tpIOP compared with GAT [25]. Their logic was that changes in the biomechanical properties of the cornea influence IOP using GAT but are less visible in transpalpebral IOP measured by Diaton. iCare measures IOP using a rebound tonometer directed at the cornea; therefore, a similar influence of changes in the cornea could affect IOP. Thus, when tpIOP was measured by Diaton to screen and monitor IOP post-refractive surgery, one should note the CCT before interpreting IOP.

One month after TPRK, the difference in IOP was greater in older patients than in younger patients in our study. It seems that the stabilization of the corneal stroma after TPRK is better in younger than in older patients. The changes in collagen crimp in both the cornea and the sclera were noted in older patients, making these tissue stiff [28]. Because age was not correlated with the difference in IOP using the two tonometers before TPRK and at W1, its significant influence one month after the TPRK study is difficult to explain. We recommend further studies to understand this correlation between age and IOP difference.

In our study, the severity of myopia did not significantly influence the difference in IOP measured by the two tonometers before and after TPRK. A study in a European country showed that post-LASIK surgery, the reduction in IOP measured by a noncontact tonometer was linked to the reduction in myopia. A one-diopter reduction following surgery resulted in 1 mmHg IOP reduction [29]. Thus, linking myopia severity to different tonometers may explain the difference in IOP by the two tonometers after surgery. However, the difference in IOP using two tonometers before surgery could be an interesting and independent issue and could have been linked to variations in CCT in different grades of myopia.

There were few limitations in our study. Because this was a cross-sectional study, IOP measured by two methods in the same eye was reviewed through randomization, and there was an adequate time gap between the measurement of IOP by two types of tonometers. One tonometer influencing the measurement of the subsequent tonometer cannot be completely ruled out. Validity parameters could be better tested using randomized clinical trial instead of cross-sectional method for more reliable evidence. The depth of corneal stromal ablation was based on CCT and the grade of myopia in the eye before surgery. Hence, IOP post-TPRK can be an effect of independent factors, as well as the extent of ablation during the intervention procedure. Thus, the influence of CCT on IOP post-TPRK could be overestimated in such analysis.

## Conclusions

In a large institute, arrays of tonometers are available to eye-care professionals. The current study shows that there could be variations in IOP measured by the types of tonometers that are based on different principles and the influence of corneal properties. Therefore, although the Diaton or iCare tonometer could be useful in screening and monitoring IOP post-refractive surgery, using the same tonometer before and after surgery could minimize spurious changes in IOP post-TPRK. IOP measured by iCare and Diaton was similar in three-fourths of the eyes. However, IOP was overestimated in a few eyes and underestimated in a few eyes measured by these two tonometers. CCT was a significant predictor of differences in IOP measured by these two methods.
